# No clear influence of preference bias on satisfaction and early functional outcome in resurfacing hip arthroplasty

**DOI:** 10.3109/17453674.2011.566140

**Published:** 2011-04-05

**Authors:** Pepijn Bisseling, José MH Smolders, Annemiek Hol, Job LC van Susante

**Affiliations:** Department of Orthopaedics, Rijnstate Hospital, Arnhem, the Netherlands

## Abstract

**Background and purpose:**

Hip resurfacing arthroplasty (RHA) is done in patients who often have a high preference for the method. This preference can influence the clinical outcome and satisfaction. We evaluated the potential influence of this preference bias.

**Patients and methods:**

From an ongoing randomized trial comparing RHA with total hip arthroplasty, 28 consecutive patients (28 hips) who had been allocated to an RHA were characterized as the “randomized” group. 22 other patients (24 hips) who had refused participation and had especially requested an RHA were characterized as the “preference” group. Harris hip score (HHS), Oxford hip score (OHS), University of California at Los Angeles activity scale (UCLA), Short Form 12 (SF-12), and visual analog scale satisfaction score (VAS) were assessed in both groups.

**Results:**

Both groups had a high implant satisfaction score (97/100 for the “preference” group and 93/100 for the “randomized” group) at 12 months. The HHS, OHS, and UCLA were similar at baseline and also revealed a similar improvement up to 12 months (p < 0.001). Regarding the SF-12, the “preference” group scored lower on the mental subscale preoperatively (p = 0.03), and there was a greater increase after 12 months (p = 0.03).

**Interpretation:**

We could not show that there was any influence of preference on satisfaction with the implant and early clinical outcome in patients who underwent RHA. The difference in mental subscale scores between groups may still indicate a difference in psychological profile.

The outcome of any surgical treatment is influenced by several factors. Apart from the surgical intervention itself, co-morbidities and postoperative rehabilitation—and also factors such as patients' perception, confidence, and expectations—contribute to the final result and patient satisfaction. Nowadays, most patients have access to the internet and other sources of information, and are well-informed. Their conceptions will lead beliefs and expectations, which will in turn lead to preferences. Preference for a specific treatment can influence the outcome (Torgerson et al. 1996, McPherson et al. 1997, Van der Windt et al 2000, McPherson and Britton 2001, Thomas et al. 2004, Klaber Moffett et al. 2005) and can introduce bias into assessments of satisfaction and acceptability. This might be a confounding factor in a trial, and may affect the validity of the results. To obtain hard evidence of any possible preference effects is problematic, as it is difficult to reliably distinguish between simple therapeutic effects and preference effects mediated through psychological pathways in experiments (McPherson and Chalmers 1998).

The dilemma of a possible influence of preference is frequently encountered in studies in orthopedic surgery. For example, the interest in resurfacing hip arthroplasty (RHA) has grown in the past 15 years (National Joint Registry for England and Wales 2004, Chen et al. 2009, National Joint Registry for England and Wales 2009) and has received much international attention. The results reported regarding the short-term and long-term follow-up of RHA appear to correspond with the results of conventional total hip arthroplasty (THA) (Pollard et al. 2006, Vail et al. 2006, Amstutz et al. 2007, Khan et al. 2009, Mont et al. 2009, Lavigne et al. 2010) and the satisfaction rates reported have been 90–100% (Khan et al. 2009, Lingard et al. 2009, Mont et al. 2009). Hip resurfacing surgeons generally deal with patients with a profound preference for this particular implant. We have not found any studies that have incorporated the possible influence of preference of the patient for an RHA into their results, and it can be speculated whether these results are influenced by this preference and perception on the part of the patients.

In an ongoing randomized trial comparing RHA with conventional THA, we encountered—as expected—some difficulty in recruiting patients for inclusion, since several patients had a specific demand for RHA. In this way, RHAs were performed in 2 groups of patients: (1) an unbiased “randomized” group without any preferences, willing to participate in the ongoing trial, and simply allocated to RHA; and (2) a “preference” group of “potentially biased” patients with a specific demand for RHA and who declined participation in the trial.We could therefore evaluate the potential role of preference bias on implant satisfaction and early clinical outcome. We hypothesized that patients in the “preference” group would be more satisfied than the patients in the “randomized” group. On the other hand, patients with a high degree of preference could have such high expectations of the treatment that they might be difficult to fulfill, which would lead to lower satisfaction compared to patients without any preference.

## Patients and methods

From April 2007 through March 2010, patients under 65 years with primary arthritis of the hip were evaluated for eligibility to enter the randomized controlled trial (RCT) comparing RHA with THA. After having given informed consent, patients with a strong preference for RHA (and who were therefore unwilling to be randomized) entered the prospective cohort study—the “preference” group. Patients with no preference were enrolled in the RCT to receive either an RHA or a THA. The current study included all patients in the “preference” group and all patients in the RCT who were allocated to RHA, with a minimum follow-up of 6 months.

The criteria for inclusion in both the RCT and the cohort were identical: patients between 35 and 65 years old, eligible for primary hip replacement because of osteoarthritis, congenital hip dysplasia, or posttraumatic arthritis. Patients were excluded in case of (previous) infection of the hip, hip fracture, avascular necrosis with collapse, osteoporotic bone mineral density index levels of the involved hip (t-score < 2.5), renal failure, or hip revision of the primary index procedure.

All patients received a Conserve Plus RHA (Wright Medical Technology, Arlington, TN). The operations were performed through a standard posterolateral approach by a senior hip surgeon with considerable experience in RHA implants (Witjes et al. 2009). Both groups received identical antibiotic prophylaxis, periarticular ossification prophylaxis, and thrombosis prophylaxis during hospital admission, and 6 weeks afterwards. The patients had identical rehabilitation protocols with unrestricted weight bearing according to individual tolerance, starting on the first postoperative day.

**Figure 1.**
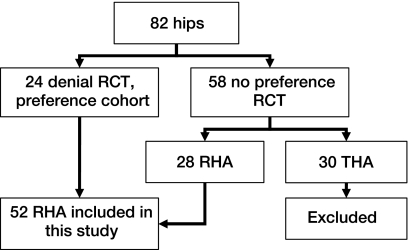
Recruitment of patients to the study.

50 patients were included in the study, with 28 implants (28 patients) in the “randomized” group and 24 implants (22 patients) in the “preference” group (Figure 1 and Table). All patients in the “preference” group and 22 of the 28 patients in the “randomized” group completed the follow-up term of 12 months. The remaining 6 patients had a follow-up of 6 months.

**Table:** Demographics of patients

	“Preference” group (n = 24)	“Randomized” group (n = 28)	p-value
Age			
median	52	58	
interquartile range	48–56	52–62	0.01
Sex: Male	15	13	0.2
Diagnosis			
Osteoarthritis	24	26	
Hip dysplasia	–	1	
Avascular necrosis	–	1	0.5

All patients completed a questionnaire that included the Short Form 12 (SF-12) and Oxford hip score (OHS) preoperatively, at 6 months, and at 12 months. The Harris hip score (HHS) and the University of California at Los Angeles activity scale (UCLA) were assessed by an independent member of the research staff (AH) who collected and registered all the forms. Satisfaction with the implant was measured on a numeric scale (visual analog scale satisfaction score (VAS)) of 0–100 mm, where 100 mm corresponded to being completely satisfied.

Approval for the randomized clinical trial and the cohort follow-up was obtained from the regional ethics committee of the Radboud University Nijmegen Medical Centre, with issue number LTC 419-071206 and date of approval 01/02/2007. All patients agreed to sign an informed consent document. The EudraCT number asigned to the randomized controlled trial was 2006-005610-12.

### Statistics

Variables were checked for normal distribution with the Shapiro-Wilk test. A value of < 0.05 was defined as the absence of a normal distribution. The mean and standard deviation (SD) were used for normally distributed variables and the median and interquartile range (IQR) for variables without normal distribution. Differences between the groups were determined by the Student's t-test for variables with normal distribution, the Mann-Whitney test for variables without normal distribution, and the Pearson Chi-square test for categorical variables (sex and diagnosis). Variables that were not normally distributed were: age, blood loss, the preoperative OHS and UCLA scores, the VAS satisfaction score at 12 months, and the change in satisfaction score between 6 and 12 months. These p-values are marked with the superscript β. Significance was defined as p-values of < 0.05. SPSS software version 15.0 was used for statistical analysis.

## Results

The characteristics of the patients are given in Table 1. Both groups were similar regarding sex and diagnosis, but the patients in the “preference” group were younger than in the “randomized” group. Mean operation time (“preference” group: 81 min (SD 15); “randomized” group: 76 min (SD 11); p = 0.2) and median blood loss (“preference” group: 300 (288–313) mL; “randomized” group: 300 (200–300) mL; p = 0.4^β^) were similar in both groups. Similar implants sizes were used in both groups (p = 0.7).

**Figure 2.**
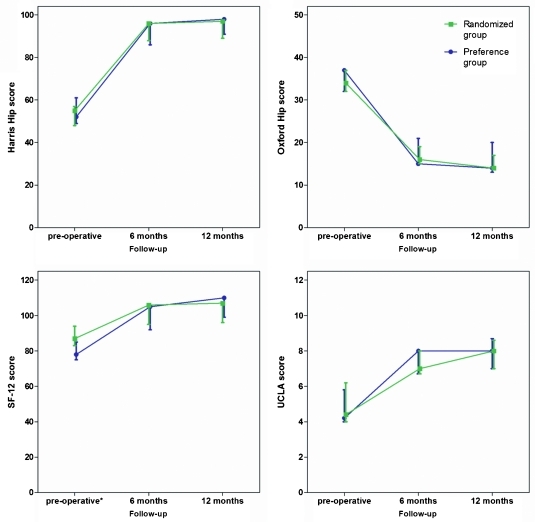
Clinical scores (HHS, Oxford, SF-12 and UCLA) with 95%-confidence interval preoperatively, at 6 and 12 months. * In the horizontal axis of the SF-12 score represents a significant difference at baseline preoperative scores (p<0.05).

The preoperative HHS, OHS, and UCLA scores were similar in both groups (Figure 2). The SF-12 score, however, was higher (88 (SD 14)), in the “randomized” group than in the “preference” group (80 (SD 12)) (p = 0.03). This difference mainly originated from intergroup differences in the mental subscale. A mean score of 47 (SD 13) on the mental subscale was found in the “preference” group, as opposed to 53 (SD 10) in the “randomized” group (p = 0.05).

The HHS, OHS, and UCLA scores all showed a postoperative improvement at 12 months compared to the preoperative baseline scores for both groups (p < 0.001) (Figure 2). These improvements were similar between the groups (p = 0.8, p = 0.7, and p = 0.4, respectively). For the SF-12, however, at 12 months a better recovery was achieved from preoperative levels in the “preference” group than in the “randomized” group (p = 0.03).

Patient satisfaction (VAS) was assessed at 6 and 12 months for both groups. Both groups had a high satisfaction score, with a median of 97 for the “preference” group and 93 for the “randomized” group at the 12-month follow-up (p = 0.7^β^). Similar scores were obtained at the 6-month follow-up.

2 complications occurred in the “preference” group. 1 patient had a perioperative collum fissure with a delayed, but uneventful, recovery—and with clinical and satisfaction scores that matched within the interquartile range. Another patient had complaints of possible anterior impingement of the RHA. This patient had clinical and satisfaction scores that dropped below the interquartile range. With exclusion of both patients, the median satisfaction score remained at 97 for the “preference” group and 93 for the “randomized” group (p = 0.6^β^). With this exclusion, there were minimal changes in clinical scores, but with no consequences for the differences between the “preference” group and the “randomized” group (p = 1.0, p = 0.9, p = 0.3, and p = 0.01 for HHS, OHS, UCLA, and SF-12, respectively). The other 50 RHAs all had an uneventful clinical course.

## Discussion

In this prospective comparative study, patient satisfaction and early clinical outcome in “biased” patients with a high preference for resurfacing hip arthroplasty (the “preference” group) did not differ statistically significantly from the results in unbiased patients who were simply allocated to an RHA after randomization in a separate randomized controlled trial (the “randomized” group). There was, however, a trend toward better satisfaction in the “preference” group. Only for the preoperative SF-12 values, and for the mental subscale in particular, was any statistically significant difference between groups encountered, in favor of the “randomized” group.

In spite of the fact that the potential bias from treatment preferences is a well-recognized phenomenon in orthopedic practice, there have only been a few studies dealing with this clinical dilemma. Van der Windt et al. (2000), for example, demonstrated a success rate of 85% in patients with shoulder pain who received their preferred therapy compared to a 64% success for those who underwent the same treatment against their preference. In another study (Thomas et al. 2004), any direct influence of preference for a certain therapy on shoulder pain could not be confirmed; however, the authors revealed that in general patients with a preference before randomization tended to have a better overall outcome than those with no preference.

Randomized controlled trials are usually regarded as the gold standard in comparing 2 therapeutic treatments, as they diminish possible confounding factors. To study the potential influence of preference bias on the outcome of one and the same surgical procedure, randomization is, however, not a feasible tool for obvious reasons. Our randomized controlled trial on THA and RHA confirmed for us the existence of patient preference for RHA; it was difficult to recruit patients for the trial. Many patients had a preference for RHA even after being informed about the absence of any evidence in the literature of a benefit of RHA over a conventional THA (Mont et al. 2009, Lavigne et al. 2010). The presence of a cohort of patients with a clear preference for RHA and a group of patients allocated to RHA after randomization enabled us to gain some insight into the possible role of preference bias.

Our study had some limitations, however. The number of patients in both groups was small, eventually resulting in a power of 59% to detect a clinical significant difference of 10 on the VAS for patient satisfaction in a post hoc power analysis. A power of 80% was calculated to detect a difference of 13 on the VAS for patient satisfaction. Clearly, there was a small difference in outcome between the groups and a larger number of patients may eventually have revealed a statistically significant difference in patient satisfaction between the groups. On the other hand, the power in our study was substantial enough for us to question whether such a difference would have been of clinical importance.

Another limitation of our study may have been the short follow-up. However, Khan et al. (2009) evaluated the Birmingham hip arthroplasty in a 5–8-year follow-up and demonstrated that the satisfaction rate did not change substantially after the first postoperative year. Lingard et al. (2009) also showed a ceiling effect after 1 year. In addition, it is debatable whether a potential difference in satisfaction after 1 year would be influenced by preference, because expectations would be most manifest in the short period after the operation.

2 patients with a bilateral prosthesis were included. One must assume that the outcome of 2 prostheses in the one patient cannot be interpreted independently. The result of the first prosthesis can either positively or negatively influence the outcome of the second, and vice versa (Bryant et al. 2006). Study outcome in general may be biased by this phenomenon; however, the number of bilateral prostheses in our study was low and exclusion of the 2 patients with bilateral prostheses did not have any consequences for our findings (data not shown).

Apart from the presence or absence of a profound preference for an RHA, both groups matched regarding most demographic features and preoperative functional scores. The size of the femoral component of the implant was similar in both groups. This is important, as component size is known to influence the outcome of RHA (Crowninshield et al. 2004, Prosser et al. 2010).

The only differences between the groups were age and preoperative SF-12 score. The difference in age between the groups suggests that younger patients are less willing to participate in a randomized clinical trial. As for the SF-12, and for the mental subscale in particular, patients in the “preference” group had a lower preoperative score. There were no outliers that could explain this difference between the groups. One could argue whether there is reason to believe that patients with a high preference for a certain treatment generally have a different psychological profile than patients who are willing to participate in a randomized trial. This finding has been recognized before (McPherson et al. 1997).

In conclusion, we could not demonstrate any influence of preference on implant satisfaction and early clinical outcome in patients with an RHA. A trend towards a relatively higher degree of satisfaction was nevertheless established for patients with a specific request for RHA. The significant difference in mental subscale scores encountered between groups may indicate a difference in psychological profile.
